# The proteomic architecture of schizophrenia iPSC-derived cerebral organoids reveals alterations in GWAS and neuronal development factors

**DOI:** 10.1038/s41398-021-01664-5

**Published:** 2021-10-19

**Authors:** Michael Notaras, Aiman Lodhi, Haoyun Fang, David Greening, Dilek Colak

**Affiliations:** 1 Center for Neurogenetics, Feil Family Brain and Mind Research Institute, Weill Cornell Medical College, Cornell University, New York, NY USA; 2 Baker Institute for Heart and Diabetes, Melbourne, VIC Australia; 3 La Trobe Institute for Molecular Science, La Trobe University, Melbourne, VIC Australia; 4 Central Clinical School, Monash University, Melbourne, VIC Australia; 5 Baker Department of Cardiometabolic Health, University of Melbourne, Melbourne, VIC Australia; 6 Gale and Ira Drukier Institute for Children’s Health, Weill Cornell Medical College, Cornell University, New York, NY USA

**Keywords:** Neuroscience, Schizophrenia

## Abstract

Schizophrenia (Scz) is a brain disorder that has a typical onset in early adulthood but otherwise maintains unknown disease origins. Unfortunately, little progress has been made in understanding the molecular mechanisms underlying neurodevelopment of Scz due to ethical and technical limitations in accessing developing human brain tissue. To overcome this challenge, we have previously utilized patient-derived Induced Pluripotent Stem Cells (iPSCs) to generate self-developing, self-maturating, and self-organizing 3D brain-like tissue known as cerebral organoids. As a continuation of this prior work, here we provide an architectural map of the developing Scz organoid proteome. Utilizing iPSCs from *n* = 25 human donors (*n* = 8 healthy Ctrl donors, and *n* = 17 Scz patients), we generated 3D cerebral organoids, employed 16-plex isobaric sample-barcoding chemistry, and simultaneously subjected samples to comprehensive high-throughput liquid-chromatography/mass-spectrometry (LC/MS) quantitative proteomics. Of 3,705 proteins identified by high-throughput proteomic profiling, we identified that just ~2.62% of the organoid global proteomic landscape was differentially regulated in Scz organoids. In sum, just 43 proteins were up-regulated and 54 were down-regulated in Scz patient-derived organoids. Notably, a range of neuronal factors were depleted in Scz organoids (e.g., MAP2, TUBB3, SV2A, GAP43, CRABP1, NCAM1 etc.). Based on global enrichment analysis, alterations in key pathways that regulate nervous system development (e.g., axonogenesis, axon development, axon guidance, morphogenesis pathways regulating neuronal differentiation, as well as substantia nigra development) were perturbed in Scz patient-derived organoids. We also identified prominent alterations in two novel GWAS factors, Pleiotrophin (PTN) and Podocalyxin (PODXL), in Scz organoids. In sum, this work serves as both a report and a resource that researchers can leverage to compare, contrast, or orthogonally validate Scz factors and pathways identified in observational clinical studies and other model systems.

## Introduction

Schizophrenia (Scz) is a debilitating brain disorder that occurs in approximately ~1% of the population [1]. While Scz onset typically occurs in early adulthood, subtle brain changes and symptoms often begin emerging years prior to onset during the so-called “prodromal period” [2, 3]. In spite of this, it has remained unclear when Scz neuropathology actually begins to unfold in the brain [1]. For instance, does Scz neuropathology begin a couple of years prior to onset in adolescence when prodromal features progressively emerge? Or does Scz neuropathology begin much earlier in neurodevelopment at a scale that is not yet resolvable? Following decades of investigation, there is now strong epidemiological evidence that indicates risk of Scz may begin to accumulate during *in utero* brain development [4–7]. This includes data from numerous, independent, large-scale population studies [4–7]. Critically, it remains unclear if *in utero* risk factors for later Scz onset, such as maternal immune activation, famine, or hormonal/steroid factors, elicit risk by inducing neurodevelopmental alterations or promoting rates of *de novo* mutation [8]. While the latter can’t be ruled out as a potential etiological contributor, the former hypothesis holds strong merit given the highly-regulated nature of cortical development *in utero* and the fact that innumerous Scz risk factors exhibit known roles in central nervous system development. Indeed, some novel biological intermediaries are starting to be discovered which link *in utero* environmental risk factors to potential genetic factors, alterations, and/or vulnerabilities [9]. However, resolving these neurodevelopmental hypotheses of Scz has been difficult. Critically, ethical and technical constraints in accessing human primary brain tissue have arrested progress in delineating the neurodevelopmental trajectory of Scz. These ethical and technical limitations are further compounded by our inability to identify prospective cases of Scz, which has further sequestered our understanding of neurodevelopmental mechanisms of psychosis and has caused a rift between the known epidemiology and the presumed neurobiology of Scz. For instance, in the largest GWAS conducted to date a total of 108 loci of risk were identified – yet, many of these loci (e.g. PTN or PODXL) had unknown disease relevance as well as ambiguously defined neurobiology. Without a means to dissect these factors in human-derived tissue, it is possible that identifying the molecular mediators underlying the ontogeny of disease onset in Scz may continue to be protracted.

Recently, we attempted to overcome these technical and ethical limitations by generating human-derived tissue using stem cells. Namely, we modeled the neurodevelopmental pathology of Scz by harnessing human induced pluripotent stem cells (iPSCs) from healthy adults (Ctrls) and idiopathic Scz patients to generate 3D brain-like tissue known as “cerebral organoids” [1]. Cerebral organoids allow human-specific mechanisms of neural development to be studied while capturing the entirety of the molecular-genetic background of patients. This is a particularly useful model system with respect to “black box” diseases such as Scz, whose neurodevelopmental origins have remained unclear, as it allows self-organizing and self-maturing human neural tissue to be spontaneously generated. Thus, 3D stem cell derived methodologies provide access to a limitless supply of human-derived tissue which can be used to dissect complex diseases defined by “*daunting polygenicity*” [10] under controlled laboratory conditions [11]. Cerebral organoids mimic trimester 1 of early brain development and putatively recapitulate the epigenetic [12], transcriptomic [13, 14], and proteomic [1, 11] architecture that is expected of the developing mammalian brain. This also includes the recapitulation of cortical cell-type diversity and cellular events such as migration [15] and evolutionary mechanisms that support neocortical neurogenesis [16]. Because of this, cerebral organoids have already been used to model prenatal drug/narcotic effects [11], microcephaly [17], macrocephaly [18], Zika virus effects [19, 20], features of autism [21–23], microdeletion syndromes [24] including 22q11 deletion syndrome [25], hypoxic injury [26], and novel neuropathology of Scz [1, 27–31]. In the case of the latter, Scz-related organoid models have revealed a range of novel phenotypes that may be associated with early neurodevelopmental alterations. This includes diminished responses to electrophysiological stimulation and depolarization [27], alterations in growth factor pathways (e.g., FGFR1 [28] including neurotrophic growth factors and their receptors in Scz progenitors and neurons [1]), immune-related alterations (e.g., TNFα [29] and IFITM3 as well as IL6ST in Scz neurons [1]), potential developmental effects in excitation and inhibition [30], and DISC1 effects on neurodevelopment [31, 32]. Recently, we added to this developing literature by being the first to discover that Scz neuropathology is encoded on a cell-by-cell basis and is defined by multiple novel mechanisms in Scz patient-derived organoids [1]. However, we have also predicted that further mechanisms related to neurodevelopment of Scz remain to be discovered [1], thus requiring deeper analysis in larger samples and populations.

Here we sought to expand our existing knowledge of Scz by providing a deep, unbiased, analysis of molecular factors regulating central nervous system development in human-derived 3D tissue. To do this, we generated cerebral organoids from a relatively large pool of human donors (*n* = 25; *n* = 8 Ctrl donors and *n* = 17 Scz donors) and adapted cutting-edge isobaric barcoding chemistry so that samples could be condensed and analytically deconstructed simultaneously via liquid-chromatography/mass-spectrometry (LC/MS). This yielded a large dataset that we have made freely available for other human, mouse, and cellular researchers to analyze. Notably, here we emphasize large-scale changes identified in this dataset, which included a broad reduction in neuronal molecules important for neural cell-type identity and development as well as metabolic and novel GWAS factors. This work and dataset may thus provide insight for other researchers and labs that have an interest in biological data from human-derived 3D stem cell systems but otherwise employ or use other model systems.

## Results

**Fig. 1:**
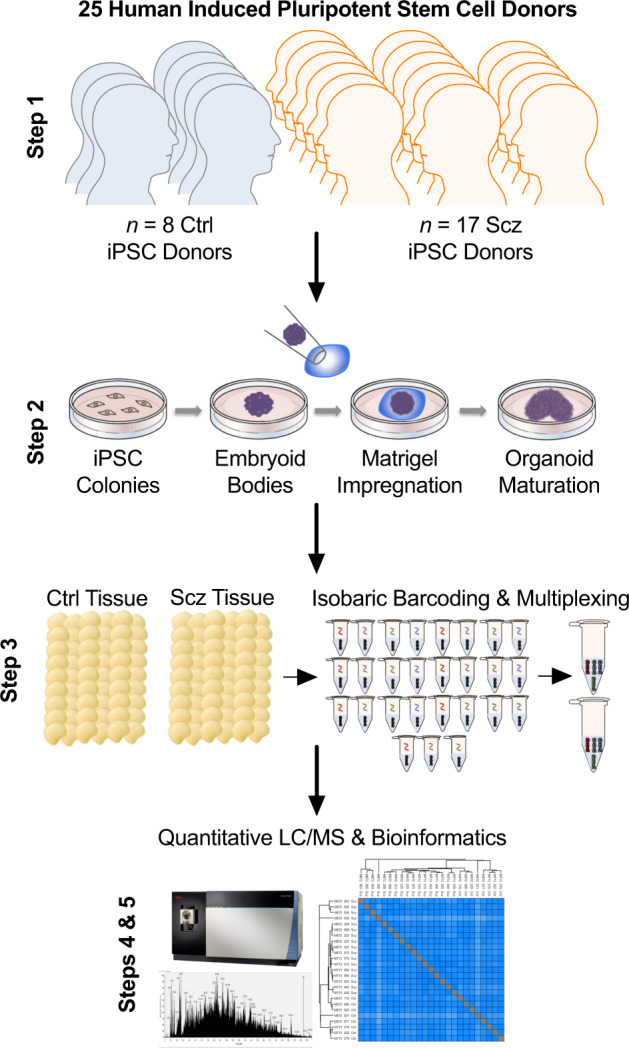
Schematic of cerebral organoid and TMT-LC/MS analytical pipeline. Briefly, 25 distinct human iPSCs were obtained from both healthy Control (Ctrl) donors and Schizophrenia (Scz) patients. Each line represented a biologically unique sample from a specific individual, and lines were predominantly obtained from NIH repositories. Following this, iPSCs were expanded and utilized to generate patient-derived cerebral organoids that mimic the 1^st^ trimester of brain assembly (see Methods, [17, 87] for protocol information, and [1] for our previous application of 3D Scz patient-derived organoids). This process involved dissociating iPSC colonies to generate 3D embryoid body aggregates that could be pushed towards a neural fate via chemically minimalist media cocktails [17, 87]. Following neural induction, organoids were implanted into a matrigel droplet as a scaffold to support tissue expansion and (broadly consistent with our prior study [54]) maturated to a primary endpoint of 35-40 DIV. Following this, samples were individually subjected to protein lysis and tryptic-based enzymatic digestion. For proteomic analysis of cerebral organoids, peptides were isobarically barcoded using TMTpro 16-Plex chemistry that allowed samples to be multiplexed for simultaneous detection of different samples via liquid chomatography–mass spectrometry (LC/MS). This allowed up to 15 samples (+1 pool) to be condensed into a single tube for simultaneous detection via LC/MS analysis, resulting in a total of 27 samples (*n* = 25 human donor organoids, + *n* = 2 internal reference pools). Bioinformatics were subsequently conducted in accordance with the parameters described in our Methods as well as two prior manuscripts that have incorporated LC/MS proteomic analysis of human-derived organoid samples [1, 11].

To study the molecular architecture of developing human brain-like tissue, we generated 3D cerebral organoids from human iPSC donors banked by the NIMH. In sum, biologics from *n* = 25 human donors were sampled comprising *n* = 8 healthy Ctrls and *n* = 17 Scz patients. Briefly, iPSCs from human donors were grown in 2D culture atop vitronectin-coated plates before being dissociated with Accutase to yield single-cell iPSC suspensions. Stem cell suspensions were correspondingly cultured into 3D aggregates, known as embryoid bodies, before being subjected to a chemically minimalist neural induction media for up to 7 days in vitro (DIV). After exhibiting evidence of neuroepithelial expansions and/or other morphological evidence of neural induction, tissue was impregnated into a matrigel droplet as a scaffold for further tissue expansion. Developing organoids were then maturated under constant agitation atop an orbital shaker. Following this, at approximately 35-40 DIV, organoids from all 25 human donors were sampled for TMT quantitative proteomics. Briefly, this involved dissociating organoids, preparing peptide suspensions (digestion, reduction, and alkylation), barcoding samples with isobaric TMTpro 16-plex chemistry, and then multiplexing samples for simultaneous detection and analysis via nano high-sensitivity proteome profiling (for a simplified schematic of our experimental pipeline, see Fig. 1).

Analysis of organoid proteomes revealed sufficient peptide coverage for high-confidence quantitative analysis of 3705 proteins (peptide >1; intensity >0) across all 25 human donor samples. Based on Log2 transformed protein intensities, the Coefficient of Variation (CV) of Scz and Ctrl proteome groups was highly stringent; Median CV for Ctrls was 1.07% and for Scz 1.23%. This provided confidence in both the degree of neural induction achieved between samples, and that organoids were overall of a very similar and thus comparable composition between iPSC donors and within groups.

To gain insight into differences between Scz and Ctrl organoids, we next sought to determine which proteins (based on their expression) differed between these groups. Further analysis revealed the significant differential expression of peptide fragments belonging to 97 proteins in Scz organoids, of which 43 were up-regulated (*p* value < 0.05, Log2FC > 0.05) and 54 were down-regulated (*p* value < 0.05, Log2FC < −0.05). Thus, in sum, ~2.62% of the total organoid proteome was differentially expressed in Scz organoids, with equivalent (~1.16% vs. ~1.46%) proportions of differentially expressed proteins being up- and down-regulated, respectively.

**Fig. 2:**
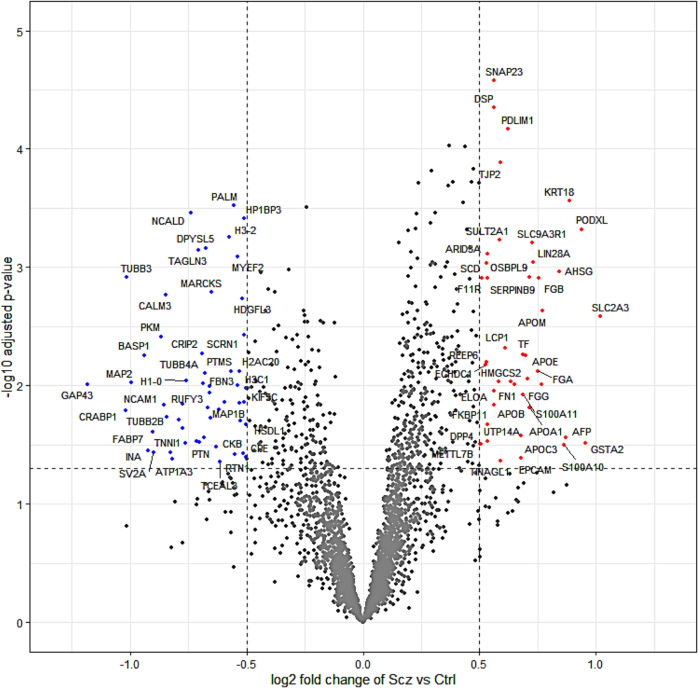
Differential expression in the Scz cerebral organoid proteome. Principal component analysis of the cerebral organoid proteome indicated data grouping based on phenotype, and protein expression distributions indicated data correlation across all samples. This statistical baseline allowed us to consider the differentially expressed proteins present in Scz patient-derived cerebral organoids, which are shown here as a volcano plot split by log2 fold change and –log10 adjusted *p* values. In sum, ~2.62% of 3705 proteins (peptide >1; intensity >0) identified exhibited differential expression. Significantly up-regulated proteins that surpassed log2 fold change thresholding are depicted to the right in red (*p* value <0.05, Log2FC > 0.05), whereas down-regulated proteins (*p* value < 0.05, Log2FC < -0.05) are presented to the left of the plot in blue. Notable Scz GWAS factors (see 108 loci identified in [33]) included the up-regulation of PODXL and down-regulation of PTN, which replicated our previous findings in a smaller cohort [1]. Note also the down-regulation of the neural stem cell proliferation factor CRABP1 [93] as well as canonical neuronal development markers (e.g. NCAM1 [94], NCALD [95], and CPE [78]), neuronal markers (e.g. MAP2, TUBB3, MAP1B), synaptic markers (e.g., SV2A). Conversely, a range of apolipoproteins (APOE, APOA1, APOB, APOC3) were found to be up-regulated in Scz patient-derived cerebral organoids.

Deeper examination of significantly down-regulated proteins in Scz organoids, sorted by Log2FC values (see Table 1), revealed several important changes. Notably, we detected a depletion of factors that support neuronal development, differentiation, identity and/or function. Down-regulated neuronal development factors in Scz organoids comprised Neuromodulin (GAP43; Log2FC = −1.183, *p* = 0.010), Cellular Retinoic Acid-Binding Protein 1 (CRABP1; Log2FC = −1.018, *p* = 0.016), Neural Cell Adhesion Molecule (NCAM1; Log2FC = −0.854, *p* < 0.014), and expression of the myelin-modulating factor Myelin Expression Factor 2 (MYEF2; Log2FC = −0.537, *p* < 0.001). Likewise, down-regulated expression of several other neuronal factors – involved in both neuronal identity and prototypic function – included Microtubule-Associated Protein 2 (MAP2), Tubulin Beta-3 Chain (TUBB3, or β3), Synaptic Vesicle Glycoprotein 2 A (SV2A), and other neuron-specific markers (see Fig. 2). In addition to these changes, we also screened our dataset against novel, yet statistically prominent, Scz GWAS factors identified in the largest population genetic dataset reported to date [33]. One important Scz GWAS factor to emerge from our analysis of down-regulated proteins in Scz organoids was Pleiotrophin (PTN). In our prior work [1], we also detected the differential expression of PTN at both the protein and RNA level in Scz organoids, including in both Scz progenitors and neurons. This better powered analysis therefore replicates this previous finding, and further establishes PTN as a potentially important Scz risk factor during early brain assembly.

**Table 1:** 54 Down-regulated proteins in Scz organoids (<−0.5 Log2FC, *p* < 0.05).

Gene Name	Protein Name	Uniprot ID	Log2FC	*P* Value
GAP43	Neuromodulin	P17677	−1.183	0.010
CRABP1	Cellular retinoic acid-binding protein 1	P29762	−1.018	0.016
TUBB3	Tubulin beta-3 chain	Q13509	−1.015	0.001
MAP2	Microtubule-associated protein 2	P11137-3	−0.996	0.009
BASP1	Brain acid soluble protein 1	P80723	−0.939	0.006
INA	Alpha-internexin	Q16352	−0.921	0.035
FABP7	Fatty acid-binding protein, brain	O15540	−0.903	0.025
SV2A	Synaptic vesicle glycoprotein 2A	Q7L0J3-2	−0.899	0.037
PKM	Pyruvate kinase PKM	P14618-2	−0.866	0.004
NCAM1	Neural Cell Adhesion Molecule	A0A087WTF6	−0.854	0.014
CALM3	Calmodulin-3	P0DP25	−0.847	0.002
TUBB2B	Tubulin beta-2B chain	Q9BVA1	−0.840	0.019
TNNI1	Troponin I 1	G3V489	−0.827	0.037
ATP1A3	Sodium/potassium-transporting ATPase subunit alpha-3	P13637	−0.817	0.042
CRMP1	Dihydropyrimidinase-related protein 1	Q14194	−0.789	0.019
RUFY3	Protein RUFY3	Q7L099	−0.774	0.014
ATAT1	Alpha-tubulin N-acetyltransferase 1	Q5SQI0-7	−0.773	0.023
PEA15	Astrocytic phosphoprotein PEA-15	Q15121	−0.764	0.030
H1-0	Histone H1.0	P07305-2	−0.760	0.009
NCALD	Neurocalcin-delta	P61601	−0.738	0.000
PPM1B	Protein phosphatase 1B	O75688	−0.714	0.030
TAGLN3	Transgelin-3	Q9UI15	−0.705	0.001
PTN	Pleiotrophin	P21246	−0.700	0.030
CRIP2	Cysteine-rich protein 2	P52943	−0.690	0.005
RAB6B	Ras-related protein Rab-6B	Q9NRW1	−0.684	0.010
ENO2	Gamma-enolase	P09104-2	−0.682	0.027
TUBB4A	Tubulin beta-4A chain	P04350	−0.676	0.008
DPYSL5	Dihydropyrimidinase-related protein 5	Q9BPU6	−0.673	0.001
SEPTIN3	Neuronal-specific septin-3	Q9UH03-2	−0.667	0.015
GDI1	Rab GDP dissociation inhibitor alpha	P31150	−0.659	0.011
FHL1	Four and a half LIM domains protein 1	Q13642-1	−0.658	0.010
TUBA1A	Tubulin alpha-1A chain	Q71U36-2	−0.653	0.019
MARCKS	Myristoylated alanine-rich C-kinase substrate	P29966	−0.650	0.002
UCHL1	Ubiquitin carboxyl-terminal hydrolase isozyme L1	P09936	−0.629	0.033
LAMA4	Laminin subunit alpha-4	Q16363-2	−0.619	0.016
TCEAL3	Transcription elongation factor A protein-like 3	Q969E4	−0.614	0.044
TUBB4B	Tubulin beta-4B chain	P68371	−0.595	0.014
H3-2	Histone HIST2H3PS2	Q5TEC6	−0.574	0.001
PTMS	Parathymosin	P20962	−0.565	0.008
PALM	Paralemmin-1	O75781-2	−0.552	0.000
RTN1	Reticulon-1	Q16799-3	−0.551	0.038
FBN3	Fibrillin-3	Q75N90	−0.538	0.010
MYEF2	Myelin Expression Factor 2	A0A087WUT0	−0.537	0.001
H2AC20	Histone H2A type 2-C	Q16777	−0.531	0.008
DPYSL2	Dihydropyrimidinase-related protein 2	Q16555	−0.529	0.014
MAP1B	Microtubule-associated protein 1B	P46821	−0.527	0.020
HDGFL3	Hepatoma-derived growth factor-related protein 3	Q9Y3E1	−0.517	0.002
CKB	Creatine kinase B-type	P12277	−0.513	0.037
KIF5C	Kinesin heavy chain isoform 5C	O60282	−0.512	0.014
SCRN1	Secernin-1	Q12765	−0.510	0.004
HP1BP3	Heterochromatin protein 1-binding protein 3	Q5SSJ5	−0.509	0.000
H3C1	Histone H3.1	P68431	−0.502	0.010
CPE	Carboxypeptidase E	D6RF88	−0.501	0.040
HSDL1	Inactive hydroxysteroid dehydrogenase-like protein 1	Q3SXM5-2	−0.501	0.021

Similar to our review of down-regulated proteins, we also identified a number of biologically interesting observations in our up-regulated Scz protein set list (see Table 2). This included up-regulation of numerous fibrinogens (FGG, FGB, FGA; Log2FC = 0.749-0.768, *p* = 0.008–0.010) and apolipoproteins (APOM, APOA1, APOE, APOC3, APOB; Log2FC = 0.562–0.771, *p* = 0.001–0.015). However, one of the most notable up-regulated protein was another Scz GWAS factor [33] that (like PTN) we had also identified in our prior Scz patient-derived organoid work [1]; namely, Podocalyxin (PODXL; Log2FC = 0.939, *p* < 0.001). Therefore, similar to our replication of down-regulated PTN expression in Scz organoids, this analysis in a larger pool of patients confirms that PODXL is another high-confidence candidate that may play a role in modulating Scz risk during early brain development.

**Table 2:** 43 Up-regulated proteins in Scz organoids (>0.5 Log2FC, *p* < 0.05).

Gene Name	Protein Name	Uniprot ID	Log2FC	*P-*value
SLC2A3	Solute carrier family 2, facilitated glucose transporter member 3	P11169	1.019	0.003
GSTA2	Glutathione S-transferase A2	P09210	0.954	0.030
PODXL	Podocalyxin	O00592-2	0.939	0.000
KRT18	Keratin, type I cytoskeletal 18	P05783	0.884	0.000
AFP	Alpha-fetoprotein	P02771	0.868	0.027
S100A10	Protein S100-A10	P60903	0.861	0.032
AHSG	Alpha-2-HS-glycoprotein	P02765	0.843	0.001
APOM	Apolipoprotein M	O95445-2	0.771	0.002
FGG	Fibrinogen gamma chain	P02679-2	0.768	0.010
FGB	Fibrinogen beta chain	P02675	0.753	0.001
FGA	Fibrinogen alpha chain	P02671-2	0.749	0.008
LIN28A	Protein lin-28 homolog A	Q9H9Z2	0.731	0.001
SLC9A3R1	Na(+)/H( + ) exchange regulatory cofactor NHE-RF1	O14745	0.726	0.001
APOA1	Apolipoprotein A-I	P02647	0.715	0.015
SERPINB9	Serpin B9	P50453	0.712	0.001
SERPINA1	Alpha-1-antitrypsin	P01009	0.705	0.009
APOE	Apolipoprotein E	P02649	0.698	0.006
TF	Serotransferrin	P02787	0.687	0.005
S100A11	Protein S100-A11	P31949	0.685	0.012
APOC3	Apolipoprotein C-III	P02656	0.678	0.027
EPCAM	Epithelial cell adhesion molecule	P16422	0.677	0.041
FN1	Fibronectin	P02751-5	0.650	0.010
APOA4	Apolipoprotein A-IV	P06727	0.634	0.009
PDLIM1	PDZ and LIM domain protein 1	O00151	0.624	0.000
LCP1	Plastin-2	P13796	0.611	0.005
TINAGL1	Tubulointerstitial nephritis antigen-like	Q9GZM7-3	0.591	0.043
TJP2	Tight junction protein ZO-2	Q9UDY2-5	0.591	0.000
SULT2A1	Sulfotransferase 2A1	Q06520	0.588	0.001
HMGCS2	Hydroxymethylglutaryl-CoA synthase, mitochondrial	P54868-2	0.580	0.009
SNAP23	Synaptosomal-associated protein 23	O00161	0.563	0.000
DSP	Desmoplakin	P15924	0.562	0.000
APOB	Apolipoprotein B-100	P04114	0.562	0.015
ELOA	Elongin-A	Q14241	0.560	0.011
UTP14A	U3 small nucleolar RNA-associated protein 14 homolog A	Q9BVJ6-3	0.536	0.029
FKBP11	Peptidyl-prolyl cis-trans isomerase FKBP11	Q9NYL4-2	0.534	0.021
F11R	Junctional adhesion molecule A	Q9Y624	0.534	0.001
ARID3A	AT-rich interactive domain-containing protein 3A	Q99856	0.532	0.001
OSBPL9	Oxysterol-binding protein-related protein 9	Q96SU4-7	0.531	0.001
REEP6	Receptor expression-enhancing protein 6	Q96HR9-2	0.530	0.006
ECHDC1	Ethylmalonyl-CoA decarboxylase	Q9NTX5-2	0.524	0.007
SCD	Acyl-CoA desaturase	O00767	0.509	0.001
METTL7B	Methyltransferase-like protein 7B	Q6UX53	0.505	0.031
DPP4	Dipeptidyl peptidase 4	P27487	0.500	0.031

We next sought to understand the potential functionality of our differentially expressed protein targets by parsing these factors into pathways, which may also unveil broader changes in regulatory networks underscoring disease-related phenotypes. We principally examined Gene Ontology (GO) pathways, parsed by annotations belonging to biological (Tables 3–4) and molecular (Tables 5–6) function of differentially expressed proteins. We first considered down-regulated GO biological pathways. Down-regulated GO biological pathways essential for normative brain assembly, development, and maturation overwhelmingly defined Scz patient-derived organoids. This included down-regulated expression of factors that map to axonogenesis, axon development, axon guidance, morphogenesis pathways regulating neuronal differentiation, and, broadly speaking, central nervous system development (due the sheer number of pathways involved here, please refer to Table 3 for statistical values). Another interesting down-regulated GO biological process pathway in Scz organoids was specific enrichment for factors regulating substantia nigra development (GO:0021762, adjusted *p* = 0.0182, Neg Log10 = 1.74), which is of interest given that this midbrain region belongs to the basal ganglia which holds broad relevance to Scz neuropathology and its treatment (e.g., dopamine and monoamine hypotheses of Scz development and symptoms). Contrary to down-regulated GO biological pathways, up-regulated pathways in Scz organoids broadly reflected pathways involved in cellular metabolism, chylomicron assembly and remodeling, sterol and steroid pathways, as well as lipoprotein remodeling and metabolism-related pathways (refer to Table 4 for statistical values).

**Table 3:** Down-regulated GO biological processes in Scz organoids (*p* < 0.05).

Biological process	GO:BP Term_ID	Adjusted *p-*value	Neg Log10 adjusted *p*
Axon Development	GO:0061564	1.88E−07	6.725
Nervous System Development	GO:0007399	2.98E−07	6.525
Plasma Membrane Bounded Cell Projection Organization	GO:0120036	7.32E−07	6.136
Axonogenesis	GO:0007409	8.26E−07	6.083
Cell Projection Organization	GO:0030030	1.16E−06	5.937
Cell Morphogenesis Involved in Neuron Differentiation	GO:0048667	1.29E−06	5.889
Neuron Projection Morphogenesis	GO:0048812	4.63E−06	5.335
Plasma Membrane Bounded Cell Projection Morphogenesis	GO:0120039	6.03E−06	5.219
Cell Projection Morphogenesis	GO:0048858	6.50E−06	5.187
Cell Part Morphogenesis	GO:0032990	8.89E−06	5.051
Cell Morphogenesis Involved in Differentiation	GO:0000904	2.09E−05	4.680
Neuron Differentiation	GO:0030182	4.18E−05	4.379
Cellular Component Morphogenesis	GO:0032989	4.21E−05	4.375
Neuron Development	GO:0048666	9.53E−05	4.021
Neuron Projection Development	GO:0031175	0.000125503	3.901
Cell Morphogenesis	GO:0000902	0.000170319	3.769
Generation of Neurons	GO:0048699	0.000192896	3.715
System Development	GO:0048731	0.000289587	3.538
Neurogenesis	GO:0022008	0.00059592	3.225
Multicellular Organism Development	GO:0007275	0.00099934	3.000
Anatomical Structure Development	GO:0048856	0.002063881	2.685
Axon Guidance	GO:0007411	0.002117016	2.674
Neuron Projection Guidance	GO:0097485	0.002173862	2.663
Negative Regulation of Microtubule Polymerization or Depolymerization	GO:0031111	0.011544881	1.938
Microtubule-Based Process	GO:0007017	0.01300057	1.886
Cytoskeleton Organization	GO:0007010	0.01330451	1.876
Anatomical Structure Morphogenesis	GO:0009653	0.014459529	1.840
Regulation of Axon Extension	GO:0030516	0.015068023	1.822
Developmental Process	GO:0032502	0.01628204	1.788
Substantia Nigra Development	GO:0021762	0.018211464	1.740
Microtubule Cytoskeleton Organization	GO:0000226	0.023833988	1.623
Regulation of Extent of Cell Growth	GO:0061387	0.027856692	1.555
Axon Extension	GO:0048675	0.047873583	1.320

**Table 4:** Up-regulated GO biological processes in Scz organoids (*p* < 0.05).

Biological process	GO:BP Term_ID	Adjusted *p* value	Neg Log10 adjusted *p*
Chylomicron Remodeling	GO:0034371	1.03E−08	7.988
Chylomicron Assembly	GO:0034378	3.76E−08	7.425
Plasma Lipoprotein Particle Assembly	GO:0034377	1.15E−07	6.938
Triglyceride-Rich Lipoprotein Particle Remodeling	GO:0034370	1.62E−07	6.790
Plasma Lipoprotein Particle Remodeling	GO:0034369	1.73E−07	6.762
Protein-Lipid Complex Remodeling	GO:0034368	1.73E−07	6.762
Protein-Containing Complex Remodeling	GO:0034367	2.53E−07	6.598
Protein-Lipid Complex Assembly	GO:0065005	2.53E−07	6.598
High-Density Lipoprotein Particle Remodeling	GO:0034375	6.89E−07	6.162
Reverse Cholesterol Transport	GO:0043691	2.10E−06	5.677
Plasma Lipoprotein Particle Organization	GO:0071827	3.08E−06	5.511
Protein-Lipid Complex Subunit Organization	GO:0071825	4.88E−06	5.312
Cholesterol Efflux	GO:0033344	1.47E−05	4.832
Terpenoid Metabolic Process	GO:0006721	1.85E−05	4.733
Very-Low-Density Lipoprotein Particle Remodeling	GO:0034372	1.97E−05	4.706
Platelet Degranulation	GO:0002576	2.31E−05	4.636
Sterol Transport	GO:0015918	2.44E−05	4.612
Phospholipid Efflux	GO:0033700	2.84E−05	4.547
Isoprenoid Metabolic Process	GO:0006720	5.53E−05	4.257
Positive Regulation of Substrate Adhesion-Dependent Cell Spreading	GO:1900026	6.58E−05	4.182
High-Density Lipoprotein Particle Assembly	GO:0034380	7.18E−05	4.144
Cell-Cell Adhesion	GO:0098609	8.60E−05	4.066
High-Density Lipoprotein Particle Clearance	GO:0034384	0.000120368	3.919
Cholesterol Homeostasis	GO:0042632	0.000156831	3.805
Post-Translational Protein Modification	GO:0043687	0.000163138	3.787
Sterol Homeostasis	GO:0055092	0.000166565	3.778
Retinoid Metabolic Process	GO:0001523	0.00024961	3.603
Regulation of Plasma Lipoprotein Particle Levels	GO:0097006	0.000263855	3.579
Regulation of Substrate Adhesion-Dependent Cell Spreading	GO:1900024	0.000344919	3.462
Diterpenoid Metabolic Process	GO:0016101	0.000345502	3.462
Cholesterol Transport	GO:0030301	0.000383496	3.416
Heterotypic Cell-Cell Adhesion	GO:0034113	0.000409755	3.387
Cholesterol Biosynthetic Process	GO:0006695	0.000568257	3.245
Secondary Alcohol Biosynthetic Process	GO:1902653	0.000568257	3.245
Regulation of Heterotypic Cell-Cell Adhesion	GO:0034114	0.000580431	3.236
Regulation of Cdc42 Protein Signal Transduction	GO:0032489	0.000667476	3.176
Sterol Biosynthetic Process	GO:0016126	0.000893117	3.049
Plasma Lipoprotein Particle Clearance	GO:0034381	0.000959187	3.018
Lipoprotein Metabolic Process	GO:0042157	0.001034387	2.985
Chylomicron Remnant Clearance	GO:0034382	0.001066283	2.972
Triglyceride-Rich Lipoprotein Particle Clearance	GO:0071830	0.001066283	2.972
Steroid Metabolic Process	GO:0008202	0.00122924	2.910
Cholesterol Metabolic Process	GO:0008203	0.001569372	2.804
Positive Regulation of Cholesterol Esterification	GO:0010873	0.001596909	2.797
Regulated Exocytosis	GO:0045055	0.00171245	2.766
Positive Regulation of Cell Morphogenesis Involved in Differentiation	GO:0010770	0.00174483	2.758
Very-Low-Density Lipoprotein Particle Clearance	GO:0034447	0.002277712	2.643
Secondary Alcohol Metabolic Process	GO:1902652	0.002312476	2.636
Homotypic Cell-Cell Adhesion	GO:0034109	0.002650901	2.577
Triglyceride Catabolic Process	GO:0019433	0.002810211	2.551
Sterol Metabolic Process	GO:0016125	0.002987054	2.525
Acylglycerol Homeostasis	GO:0055090	0.003467959	2.460
Triglyceride Homeostasis	GO:0070328	0.003467959	2.460
Lipid Homeostasis	GO:0055088	0.003686054	2.433
Vesicle-Mediated Transport	GO:0016192	0.003686287	2.433
Regulation of Triglyceride Metabolic Process	GO:0090207	0.004233532	2.373
Regulation of Cell Morphogenesis Involved in Differentiation	GO:0010769	0.004547582	2.342
Secretion	GO:0046903	0.004648136	2.333
Cell Adhesion	GO:0007155	0.00503037	2.298
Biological Adhesion	GO:0022610	0.005314483	2.275
Organic Hydroxy Compound Transport	GO:0015850	0.005357541	2.271
Intermembrane Lipid Transfer	GO:0120009	0.006131955	2.212
Exocytosis	GO:0006887	0.006499986	2.187
Steroid Biosynthetic Process	GO:0006694	0.006623642	2.179
Cdc42 Protein Signal Transduction	GO:0032488	0.006865718	2.163
Regulation of Cholesterol Ssterification	GO:0010872	0.006865718	2.163
Regulation of Triglyceride Catabolic Process	GO:0010896	0.006865718	2.163
Acylglycerol Catabolic Process	GO:0046464	0.007287829	2.137
Neutral Lipid Catabolic Process	GO:0046461	0.007287829	2.137
Substrate Adhesion-Dependent Cell Spreading	GO:0034446	0.008081591	2.093
Negative Regulation of Plasma Lipoprotein Oxidation	GO:0034445	0.008917017	2.050
Regulation of Plasma Lipoprotein Oxidation	GO:0034444	0.008917017	2.050
Secretion by Cell	GO:0032940	0.009039373	2.044
Triglyceride Metabolic Process	GO:0006641	0.00964557	2.016
Positive Regulation of Cell Adhesion	GO:0045785	0.009890818	2.005
Regulation of Cell Morphogenesis	GO:0022604	0.01018014	1.992
Positive Regulation of Heterotypic Cell-Cell Adhesion	GO:0034116	0.010529465	1.978
Regulation of Cell-Cell Adhesion	GO:0022407	0.011281044	1.948
Negative Regulation of Blood Coagulation	GO:0030195	0.01262872	1.899
Negative Regulation of Hemostasis	GO:1900047	0.013577968	1.867
Export from Cell	GO:0140352	0.014773845	1.831
Cholesterol Esterification	GO:0034435	0.015294749	1.815
Steroid Esterification	GO:0034433	0.015294749	1.815
Sterol Esterification	GO:0034434	0.015294749	1.815
Positive Regulation of Cell-Substrate Adhesion	GO:0010811	0.01579924	1.801
Negative Regulation of Coagulation	GO:0050819	0.019135052	1.718
Lipid Catabolic Process	GO:0016042	0.020745205	1.683
Platelet Aggregation	GO:0070527	0.023183729	1.635
Plasma Lipoprotein Particle Oxidation	GO:0034441	0.026712669	1.573
Acylglycerol Metabolic Process	GO:0006639	0.028359777	1.547
Neutral Lipid Metabolic Process	GO:0006638	0.029346144	1.532
Supramolecular Fiber Organization	GO:0097435	0.029625	1.528
Cell Activation	GO:0001775	0.029657104	1.528
Macromolecule Localization	GO:0033036	0.029741556	1.527
Transport	GO:0006810	0.030983148	1.509
Organic Hydroxy Compound Biosynthetic Process	GO:1901617	0.031015934	1.508
Regulation of Blood Coagulation	GO:0030193	0.033129732	1.480
Alcohol Biosynthetic Process	GO:0046165	0.035839906	1.446
Regulation of Hemostasis	GO:1900046	0.037051615	1.431
Plasminogen Activation	GO:0031639	0.037580747	1.425
Regulation of Lipoprotein Lipase Activity	GO:0051004	0.037580747	1.425
Regulation of Localization	GO:0032879	0.04005842	1.397
Glycerolipid Catabolic Process	GO:0046503	0.041304441	1.384
Vascular Process in Circulatory System	GO:0003018	0.041522396	1.382
Regulation of Vesicle-Mediated Transport	GO:0060627	0.044575903	1.351
Regulation of Cholesterol Transport	GO:0032374	0.045905316	1.338
Regulation of Sterol Transport	GO:0032371	0.045905316	1.338
Fibrinolysis	GO:0042730	0.048124122	1.318
Regulation of Coagulation	GO:0050818	0.048341707	1.316

**Table 5:** Down-regulated GO molecular functions in Scz organoids (*p* < 0.05).

Molecular function	GO:MF Term_ID	Adjusted *p*-value	Neg Log10 adjusted *p*
Structural Constituent of Cytoskeleton	GO:0005200	0.000173652	3.760
Cytoskeletal Protein Binding	GO:0008092	0.005488124	2.261
GTPase Activity	GO:0003924	0.007451524	2.128
Nucleoside-Triphosphatase Activity	GO:0017111	0.008195516	2.086
Pyrophosphatase Activity	GO:0016462	0.020438022	1.690
Hydrolase Activity, Acting on Acid Anhydrides, in Phosphorus-Containing Anhydrides	GO:0016818	0.023962071	1.620
Hydrolase Activity, Acting on Acid Anhydrides	GO:0016817	0.024306171	1.614
Tubulin Binding	GO:0015631	0.034081605	1.467
GTP Binding	GO:0005525	0.034081605	1.467
Microtubule Binding	GO:0008017	0.043893561	1.358
Structural Molecule Activity	GO:0005198	0.047624863	1.322
Guanyl Ribonucleotide Binding	GO:0032561	0.047641356	1.322
Guanyl Nucleotide Binding	GO:0019001	0.047641356	1.322

**Table 6:** Up-regulated GO molecular functions in Scz organoids (*p* < 0.05).

Molecular function	GO:MF Term_ID	Adjusted *p*-value	Neg Log10 adjusted *p*
Sterol Transporter Activity	GO:0015248	4.01E−06	5.397
Cadherin Binding Involved in Cell-Cell Adhesion	GO:0098641	2.28E−05	4.642
Cell-Cell Adhesion Mediator Activity	GO:0098632	2.68E−05	4.571
Cholesterol Transfer Activity	GO:0120020	5.42E−05	4.266
Cell Adhesion Mediator Activity	GO:0098631	6.36E−05	4.197
Sterol Transfer Activity	GO:0120015	6.55E−05	4.184
Phosphatidylcholine-Sterol O-Acyltransferase Activator Activity	GO:0060228	7.46E−05	4.127
Cell Adhesion Molecule Binding	GO:0050839	7.67E−05	4.115
Lipoprotein Particle Receptor Binding	GO:0070325	0.000150248	3.823
Lipid Transporter Activity	GO:0005319	0.000343266	3.464
Lipid Transfer Activity	GO:0120013	0.001163791	2.934
Sterol Binding	GO:0032934	0.003401382	2.468
High-Density Lipoprotein Particle Receptor Binding	GO:0070653	0.005374955	2.270
Steroid Binding	GO:0005496	0.025672825	1.591
Signaling Receptor Binding	GO:0005102	0.031882878	1.496

Broadly speaking, these changes were also reflected in our analysis of GO pathways annotated for molecular functionality. Specifically, down-regulated GO molecular functions in Scz organoids comprised cytoskeletal structural, binding, and activity, as well as metabolic pathways relevant to neurodevelopment such GTP binding and GTPase activity (see Table 5; also identified in our prior prenatal drug modeling organoid work [11]). Similarly, up-regulated GO molecular function pathways in Scz organoids were typically related to sterol activity, cell adhesion, and lipoprotein binding/transfer/activity (see Table 6). In sum, these data provide additional veracity to the idea that there are metabolic functions underscoring the depletion of neuronal development factors in Scz organoids.

Lastly, we also considered whether Reactome pathways might unveil other novel biology in Scz organoids. Overall, an analysis of down-regulated (Table 7) and up-regulated (Table 8) Reactome pathways in Scz organoids revealed broadly similar pathway enrichment to those identified via GO analysis, with some notable exceptions. First, in our down-regulated Reactome pathway analysis, we noted that there were numerous significant pathways involved in NMDA receptor activation and assembly, ER to Golgi transport, as well as synaptic transmission (see Table 7 for a comprehensive list and statistical values). Contrary to this, and in addition to a convergent detection of lipoprotein-related metabolism pathways, unique Reactome pathways that were up-regulated in Scz organoids comprised post-translational protein phosphorylation, pathways related to MAPK signaling, and IGF-related pathways. Overall, these data suggest that ying-and-yang alterations in Scz organoids exist, whereby the disruption of neuronal-development factors and pathways yields enrichment for pathways presumably involved in either compensation or other disease-related neuropathology including phenotypes that have possibly not yet been articulated in human-derived tissue (e.g. specific metabolic changes).

**Table 7:** Down-regulated reactome pathways in Scz organoids (*p* < 0.05).

Reactome pathway	Reactome Term_ID	Adjusted *p-*value	Neg Log10 adjusted *p*
L1CAM Interactions	REAC:R-HSA-373760	4.04E−07	6.393
Microtubule-Dependent Trafficking of Connexons from Golgi to the Plasma Membrane	REAC:R-HSA-190840	7.37E−07	6.133
Transport of Connexons to the Plasma Membrane	REAC:R-HSA-190872	9.65E−07	6.015
Recycling Pathway of L1	REAC:R-HSA-437239	1.35E−06	5.869
Post-Chaperonin Tubulin Folding Pathway	REAC:R-HSA-389977	2.00E−06	5.698
COPI-Independent Golgi-to-ER Retrograde Traffic	REAC:R-HSA-6811436	2.51E−06	5.601
Formation of Tubulin Folding Intermediates by CCT/TriC	REAC:R-HSA-389960	3.09E−06	5.510
Activation of AMPK Downstream of NMDARs	REAC:R-HSA-9619483	4.59E−06	5.338
Prefoldin Mediated Transfer of Substrate to CCT/TriC	REAC:R-HSA-389957	4.59E−06	5.338
Sealing of the Nuclear Rnvelope (NE) by ESCRT-III	REAC:R-HSA-9668328	9.34E−06	5.030
RHO GTPases Activate IQGAPs	REAC:R-HSA-5626467	9.34E−06	5.030
Cooperation of Prefoldin and TriC/CCT in Actin and Tubulin Folding	REAC:R-HSA-389958	1.10E−05	4.959
Gap Junction Assembly	REAC:R-HSA-190861	2.30E−05	4.638
HCMV Early Events	REAC:R-HSA-9609690	3.03E−05	4.518
Assembly and Cell Surface Presentation of NMDA Receptors	REAC:R-HSA-9609736	4.36E−05	4.360
Aggrephagy	REAC:R-HSA-9646399	4.91E−05	4.309
Carboxyterminal Post-Translational Modifications of Tubulin	REAC:R-HSA-8955332	6.18E−05	4.209
Gap Junction Trafficking	REAC:R-HSA-190828	8.54E−05	4.069
HCMV Infection	REAC:R-HSA-9609646	9.31E−05	4.031
Gap Junction Trafficking and Regulation	REAC:R-HSA-157858	9.47E−05	4.024
Intraflagellar Transport	REAC:R-HSA-5620924	0.000140206	3.853
HSP90 Chaperone Cycle for Steroid Hormone Receptors (SHR)	REAC:R-HSA-3371497	0.000184495	3.734
Kinesins	REAC:R-HSA-983189	0.000259999	3.585
Nuclear Envelope (NE) Reassembly	REAC:R-HSA-2995410	0.000519297	3.285
Translocation of SLC2A4 (GLUT4) to the Plasma Membrane	REAC:R-HSA-1445148	0.000597876	3.223
Golgi-to-ER Retrograde Transport	REAC:R-HSA-8856688	0.000664661	3.177
Axon Guidance	REAC:R-HSA-422475	0.00067359	3.172
Post NMDA Receptor Activation Events	REAC:R-HSA-438064	0.000783019	3.106
The Role of GTSE1 in G2/M Progression after G2 Checkpoint	REAC:R-HSA-8852276	0.000949362	3.023
Nervous System Development	REAC:R-HSA-9675108	0.001007002	2.997
Selective Autophagy	REAC:R-HSA-9663891	0.001010583	2.995
Activation of NMDA Receptors and Postsynaptic Events	REAC:R-HSA-442755	0.00171304	2.766
Recruitment of NuMA to Mitotic Centrosomes	REAC:R-HSA-380320	0.002242316	2.649
Chaperonin-Mediated Protein Folding	REAC:R-HSA-390466	0.002362049	2.627
Factors Involved in Megakaryocyte Development and Platelet Production	REAC:R-HSA-983231	0.002639065	2.579
COPI-Dependent Golgi-to-ER Retrograde Traffic	REAC:R-HSA-6811434	0.003037982	2.517
COPI-Mediated Anterograde Transport	REAC:R-HSA-6807878	0.003347233	2.475
Protein Folding	REAC:R-HSA-391251	0.003347233	2.475
CRMPs in Sema3A Signaling	REAC:R-HSA-399956	0.003988868	2.399
Hedgehog ‘off’ State	REAC:R-HSA-5610787	0.005515212	2.258
Neurotransmitter Receptors and Postsynaptic Signal Transmission	REAC:R-HSA-112314	0.005949918	2.225
EML4 and NUDC in Mitotic Spindle Formation	REAC:R-HSA-9648025	0.006005753	2.221
Cilium Assembly	REAC:R-HSA-5617833	0.006863941	2.163
Intra-Golgi and Retrograde Golgi-to-ER traffic	REAC:R-HSA-6811442	0.007259898	2.139
Resolution of Sister Chromatid Cohesion	REAC:R-HSA-2500257	0.008317829	2.080
MHC Class II Antigen Presentation	REAC:R-HSA-2132295	0.008317829	2.080
RHO GTPase Effectors	REAC:R-HSA-195258	0.0094632	2.024
Developmental Biology	REAC:R-HSA-1266738	0.011666055	1.933
Macroautophagy	REAC:R-HSA-1632852	0.012565891	1.901
RHO GTPases Activate Formins	REAC:R-HSA-5663220	0.013491876	1.870
Signaling by Hedgehog	REAC:R-HSA-5358351	0.02087328	1.680
Autophagy	REAC:R-HSA-9612973	0.02087328	1.680
ER to Golgi Anterograde Transport	REAC:R-HSA-199977	0.025184333	1.599
Transmission across Chemical Synapses	REAC:R-HSA-112315	0.028395357	1.547
M Phase	REAC:R-HSA-68886	0.044247896	1.354

**Table 8:** Up-regulated reactome pathways in Scz organoids (*p* < 0.05).

Reactome pathway	Reactome Term_ID	Adjusted *p-*value	Neg Log10 adjusted *p*
Post-Translational Protein Phosphorylation	REAC:R-HSA-8957275	8.31E−09	8.080
Chylomicron Assembly	REAC:R-HSA-8963888	1.82E−08	7.739
Chylomicron Remodeling	REAC:R-HSA-8963901	1.82E−08	7.739
Regulation of Insulin-like Growth Factor (IGF) Transport and Uptake by Insulin-like Growth Factor Binding Proteins (IGFBPs)	REAC:R-HSA-381426	3.18E−08	7.498
Plasma Lipoprotein Assembly	REAC:R-HSA-8963898	6.10E−07	6.215
Retinoid Metabolism and Transport	REAC:R-HSA-975634	1.25E−06	5.902
Metabolism of Fat-Soluble Vitamins	REAC:R-HSA-6806667	2.16E−06	5.665
Plasma Lipoprotein Remodeling	REAC:R-HSA-8963899	9.87E−06	5.006
Platelet Degranulation	REAC:R-HSA-114608	3.04E−05	4.517
Response to Elevated Platelet Cytosolic Ca2+	REAC:R-HSA-76005	3.98E−05	4.400
Regulation of TLR by Endogenous Ligand	REAC:R-HSA-5686938	0.000100533	3.998
Visual Phototransduction	REAC:R-HSA-2187338	0.000156361	3.806
Metabolism of Vitamins and Cofactors	REAC:R-HSA-196854	0.000455116	3.342
Plasma Lipoprotein Assembly, Remodeling, and Clearance	REAC:R-HSA-174824	0.000616778	3.210
HDL remodeling	REAC:R-HSA-8964058	0.000786847	3.104
Hemostasis	REAC:R-HSA-109582	0.001130796	2.947
GRB2:SOS Provides Linkage to MAPK Signaling for Integrins	REAC:R-HSA-354194	0.003373717	2.472
Platelet Activation, Signaling and Aggregation	REAC:R-HSA-76002	0.003972412	2.401
p130Cas Linkage to MAPK Signaling for Integrins	REAC:R-HSA-372708	0.004208218	2.376
Scavenging by Class A Receptors	REAC:R-HSA-3000480	0.008886469	2.051
Common Pathway of Fibrin Clot Formation	REAC:R-HSA-140875	0.014033493	1.853
Integrin Signaling	REAC:R-HSA-354192	0.023493017	1.629
Chylomicron Clearance	REAC:R-HSA-8964026	0.032311883	1.491
Scavenging by Class B Receptors	REAC:R-HSA-3000471	0.032311883	1.491
Integrin Cell Surface Interactions	REAC:R-HSA-216083	0.043417684	1.362
Plasma Lipoprotein Clearance	REAC:R-HSA-8964043	0.044251662	1.354

## Discussion

The aim of the current study was to further our knowledge of Scz by providing a deep, unbiased, analysis of molecular factors regulating central nervous system development in human-derived 3D tissue. To circumvent ethical and technical limitations in being able to access developing neural tissue from Scz patients [11], we generated 3D iPSC-derived cerebral organoids from *n* = 25 human donors (*n* = 8 Ctrl donors and *n* = 17 Scz donors). This approach allowed us to generate a theoretically limitless supply of self-regulating 3D neural tissue that recapitulated hallmark features of early brain assembly and corticogenesis [34, 35]. Samples were correspondingly subjected to cutting-edge isobaric barcoding chemistry that allowed up to 15 human donor samples (+ 1 pool for normalization) to be condensed into a single tube that could then be deconstructed via high-sensitivity, online, nano liquid-chromatography/mass-spectrometry proteomics. This allowed us to generate a posttranslational molecular map of factors in Scz patient-derived tissue/organoid samples. Consequently, we were able to identify that Scz organoids principally differed from healthy Ctrls due to differences in the total quantity of molecular factors (rather than their diversity), the altered expression of an ensemble of neuronal factors, and the differential regulation of specific GWAS-implicated [33] disease candidates (namely, PTN and PODXL).

### Convergence upon depletion of neuronal factors in Scz organoids

The first phenotype to arise in our molecular mapping of Scz organoids was the extent to which canonical neuron identity and development factors were depleted in Scz patient-derived organoids. For several decades, numerous theories have emerged which link neuronal and synaptic function with Scz [36–38], particularly as it relates to cortical dysfunction [39–41] and the cognitive symptoms [42, 43] observed in clinical cases [44]. Recently, progress has been made in understandingly early-arising changes within the developing brain that may influence novel neurodevelopmental factors with putative links to Scz [45]. This has led to numerous investigations of early-arising biological phenomenon in various model systems. Human-derived models, usually leveraging the power of gene edited or patient-derived iPSCs, have consequently revealed alterations in neuronal differentiation [46], mitochondrial metabolic function [47, 48], catecholamine levels [49], neuron-glia interactions [50], synaptogenesis [51], and synaptic function [52]. Thus, patient-derived iPSCs have proven to be a powerful tool in tracing early neurodevelopmental features of Scz [53]. However, iPSCs can be further exploited if used to generate human-derived organoids, a model system of human brain development which recapitulates endogenous self-regulatory mechanisms associated with cortical patterning [11]. Building upon prior Scz organoid work [1, 27–29], here we report lower levels of an ensemble of neuron-related development factors comprising GAP43, CRABP1, NCAM1, and MYEF2 as well as identity factors comprising MAP2, TUBB3, and SV2A. Broadly speaking, these molecular findings are consistent with our prior work which reported disrupted neurogenesis and lower total neuron numbers within Scz cerebral organoids [1, 54, 55] – a phenotype which has also been independently reported by other groups [28]. Thus, fewer neurons will result in less MAP2, TUBB3, and SV2A expression, which is consistent with the molecular outcomes of this independent investigation. Our detection of lower NCAM1 protein levels in Scz organoids is also consistent with a prior report that found decreased NCAM1 expression in Scz neural progenitor cells [56]. Alterations in the growth-associated factor GAP43 have also been observed across multiple brain regions and independent studies that have evaluated postmortem Scz patient tissue [57–61]. When combined, these data support the idea [1, 28] that a loss of factors which support neuronal development yields an upstream depletion of neurons within Scz patient-derived organoids [1, 28].

### Regulation of novel GWAS factors (PTN & PODXL) in Scz organoids

The other major phenotype identified in our molecular mapping of Scz cerebral organoids was the differential expression of two novel GWAS factors, namely PTN and PODXL. This analysis comprised us cross-referencing the highest-confident GWAS factors identified in unbiased clinical samples (see [33]) with our complete list of differentially expressed proteins. In our prior report utilizing a smaller TMT-LC/MS cohort design [1], we identified the differential expression of four GWAS candidates in Scz cerebral organoids at the protein level (PTN, COMT, PLCL1, and PODXL). Of these candidates, we were able to detect and replicate the differential expression of two of these factors in our much larger sample of *n* = 25 reported here. This specifically comprised alterations in PTN (down-regulated) and PODXL (up-regulated). These factors represent high-confidence GWAS factors associated with Scz, but otherwise have relatively unknown disease relevance. PTN has also been reported to be depleted in neural progenitors and shown to regulate both neurogenesis and survival phenotypes in Scz cerebral organoids [1], providing the first functional molecular data related to this candidate within the Scz literature. Other groups have also recently identified that PTN secreted from neural stem cells supports the maturation of new-born neurons [62], and can function as a neurotrophic growth factor in vivo to modulate neuronal loss [63] and long-term potentiation induction [64]. PTN has also since been implicated in a novel amphetamine-model of relevance to Scz [65], a recent computational protein-network analysis underlying Scz [66], as well as at least one nascent Scz gene-association study (*n* = 1,823 humans) [67]. On the other hand, little work has been completed on the role of PODXL in Scz, probably because PODXL is a renal-enriched factor most often associated with kidney podocytes and mesothelial cells [68]. Of note, PODXL has recently been shown to play a role in neurite outgrowth, branching, axonal fasciculation, and synapse number [69], supporting a potential role for this factor in synaptic plasticity. Additionally, PODXL was recently shown to be an apical determinant that may alter lumen size of neural progenitor cell rosettes during morphogenesis [70]. Thus, PODXL may be a fruitful target for future investigations seeking to deconvolute the role of novel Scz GWAS factors within the developing brain.

### Other novel differentially expressed candidates in Scz organoids

Lastly, it is worth emphasizing several other differentially expressed molecular candidates observed in Scz cerebral organoids hold biological interest. First and foremost, we identified that Carboxypeptidase E (CPE) was downregulated in Scz cerebral organoids. CPE is a prohormone-processing enzyme [71] and regulated secretory pathway receptor [72], possibly best known for regulating the sorting and activity-dependent secretion of BDNF [73, 74] as well as TrkB surface insertion [75] in neurons. However, CPE was recently suggested to also function as a growth factor independently of its enzymatic and sorting activities [76]. Indeed, amongst other reports suggesting a role in neuroprotection [77], it has recently been shown that CPE regulates cortical neuron migration and dendritic morphology [78]. However, the degree to which these effects is dependent upon its cargo, which includes other growth factors (e.g. BDNF), remains unclear. Lastly, the other notable differentially expressed candidates worthy of discussion comprised alterations within the apolipoprotein family, specifically APOM, APOA1, APOE, APOC3, and APOB. Apolipoproteins have been previously investigated as potential metabolic-related biomarkers [79] in peripherally accessible biological fluids (e.g. CSF [80] or plasma [81]). This specifically includes alterations in APOE and APOA1 in Scz patients [82]. These findings are broadly related to cholesterol [83], fatty acid [84], phospholipid metabolism [85], as well as other membrane-related [86] hypotheses of Scz (which are all somewhat related and/or derived from similar evidence pools). Nonetheless, it is interesting that evidence related to these hypotheses was detectable and reproducible across our sample of patients, and may indicate that further work on potential metabolic factors may also be a further avenue of fruitful research.

## Conclusion

In closing, we identified a broad reduction in molecules important for neuronal identity and development as well as specific alterations in novel GWAS and other disease-relevant molecules previously implicated in Scz. This work collectively supports the idea that Scz is a complex disease underscored by multifaceted changes that likely yield cell-specific as well as multiple mechanisms [54]. In closing, the authors hope that the current dataset may provide insight for other researchers and labs that have an interest in biological data from human-derived 3D stem cell systems but otherwise employ other model systems.

## Methods

### Induced pluripotent stem cells

Briefly, human stem cells were principally acquired from NIH deposits at the Rutgers University Cell and DNA Repository. The benefit of utilizing NIH deposited lines is that all biologics have been characterized for identity, pluripotency, exogenous reprogramming factor expression, genetic stability, and viability. In sum, we sampled a total of 25 different iPSC lines comprising both healthy Ctrls and idiopathic Scz patients. Cerebral organoids were generated from all donors in this study, and each iPSC line was biologically independent (representing a unique human donor). Ctrl iPSC lines utilized for cellular experiments included MH0159019, MH0159020, MH0159021, MH0159022, MH0167170, MH0174677, and MH0174686. One Ctrl line (GM23279) was sourced from the Coriell Institute for Medical Research. Scz iPSC lines included MH0159025, MH0159026, MH0185223, MH0185225, MH0200865, MH0217268, MH0185900, MH0185954, MH0185958, MH0185963, MH0185970, MH0185912, MH0185945, MH0185964, MH0185966, MH0185925, and MH0185928. Clinical information for Scz patients is available in Table S1 of our prior publication [1]. All Scz samples were derived from idiopathic cases, which we define here as schizophrenia cases that maintained unknown disease origins and do not meet a genetic/syndrome-based diagnosis (as listed in NIH/NIMH notes). Ctrl iPSC lines were screened for both personal, and family history, of major mental illnesses. All iPSC lines were maintained on Vitronectin-coated plates and fed with Essential 8 (E8) + E8 supplement media (ThermoFisher, CAT#: A1517001).

### 3D cerebral organoid tissue generation

We adapted the same undirected-differentiation organoid system that we used in our previous, more extensive, analysis of Scz neurodevelopmental mechanisms [1], which had been previously published by Lancaster et al. in *Nature* [17] and *Nature Protocols* [87]. Briefly, 2D iPSC colonies were dissociated and cultured into 3D embryoid bodies in ultra-low attachment plates (Corning; CAT#: 3474). Rock inhibitor (1:1000; Stem Cell Tech, CAT#: 72304) and basic fibroblast growth factor (Pepro Tech, CAT#: 100-18B) are included in media for the first 2-4 days of embryoid body culturing to promote stem cell aggregation and survival. Following this, healthy embryoid bodies are isolated and transferred to Nunclon Sphera 24 well plates (Thermo Scientific, CAT#: 174930) for neural fate specification, using neural induction media. Successful early ‘organoids’ were embedded in a 30 µl Matrigel (Corning, CAT#: 354234) spheroid-droplet and polymerized at 37 °C for 20-30 min which provided a matrix for subsequent neural expansion. Organoids suspended in matrigel droplets were next cultured in terminal organoid media for 4-6 days without agitation, and then cultured with agitation at 60-70RPM until harvested for experiments. For further organoid protocol detail, including QC steps, please refer to our previous publication [1]. Likewise, for further insight into organoid handling for proteomic analysis, please refer to our other organoid manuscript [11].

### Proteomics sample preparation, TMT labeling and LC/MS

Isobaric stable isotope labeling was achieved viaTandem Mass Tag pro (TMTpro) chemistry and Liquid-Chromatography/Mass-Spectrometry (LC/MS) proteomics as previously described [1, 11, 65]. Briefly, intact organoids were reduced with dithiotreitol and underwent alkylation with iodoacetamide before tryptic digestion at 37 °C overnight. For barcoding chemistry, we employed TMTpro 16-plex labeling according to the manufacturer’s instructions (Thermo Fisher Scientific, CAT# A44521). Each multi-plex experiment contained relevant organoid samples with an additional pooled isobaric reference label made up of the same peptide digest from the pooled mix of organoids (for data normalization between runs; TMT Tag 134 N for both TMT-LC/MS runs). A list of sample labeling strategies and replicates is available in the PRIDE proteomics exchange repository. TMT-labeled peptides were desalted using C18’ stage-tips prior to LC-MS analysis. An EASY-nLC 1200, which was coupled to a Fusion Lumos mass spectrometer, (Thermo Fisher Scientific) was utilized in positive, data-dependent acquisition mode, with samples analyzed in technical duplicate. Buffer A (0.1% FA in water) and buffer B (0.1% FA in 80% ACN) were used as mobile phases for gradient separation. TMT-labeled peptides were analyzed on a 75 μm I.D. column (ReproSil-Pur C18-AQ, 3μm, Dr. Maisch GmbH, German) was packed in-house. A separation gradient of 5–10% buffer B over 1 min, 10%-35% buffer B over 229 min, and 35%-100% B over 5 min at a flow rate of 300 nL/min was adapted. An Orbitrap mass analyzer acquired Full MS scans over a range of 350-1500 m/z with resolution 120,000 at m/z 200. The top 20 most-abundant precursors were selected with an isolation window of 0.7 Thomsons and fragmented by high-energy collisional dissociation with normalized collision energy of 40. The Orbitrap mass analyzer was also used to acquire MS/MS scans. The automatic gain control target value was 1e6 for full scans and 5e4 for MS/MS scans respectively, and the maximum ion injection time was 54 ms for both.

### Data processing and bioinformatics pipeline for quantitative analysis

Mass spectra were pre-processed as described [1, 11, 65] and processed using MaxQuant [88] (1.5.5.1). Spectra were searched against the full set of human protein sequences annotated in UniProt (sequence database Sep-2017) using Andromeda. Data was searched as described [1, 11] as a separate and single (combined) batches, with fixed modification, cysteine carbamidomethylation and variable modifications, N-acetylation and methionine oxidation. Searches were performed using a 20 ppm precursor ion tolerance for total protein level analysis. Further modifications included TMT tags on peptide N termini/lysine residues (+229.16293 Da) set as static modifications. Data was processed using trypsin/P as the proteolytic enzyme with up to 2 missed cleavage sites allowed. Peptides less than seven amino acids were not considered for further analysis because of lack of uniqueness, and a 1% False-Discovery Rate (FDR) was used to filter at peptide and protein levels. Protein identification required at least two unique or razor peptides per protein group. Contaminants, and reverse identification were excluded from further data analysis. Quantification was performed with the reporter ion quantification normalization in MaxQuant. Protein intensities were log2 transformed using Perseus [89] (1.x.10). The violin plots of log2 transformed protein intensity distribution and the boxplot of coefficient of variations per sample group were visualized using R package ggplot2. Proteins quantified in at least 70% of samples in at least one sample group were subjected to downstream visualization (principal component analysis, volcano plot) and statistical analysis using Perseus. For principal component analysis, missing values were imputed from normal distribution (downshift 1.8, width 0.3) using Perseus. For differential expression analysis proteins were subjected to Welch’s t-test; p-value < 0.05 and |log2FC | >0.5 visualized in volcano plot and subjected to downstream functional enrichment analysis using g:Profiler, including Gene Ontology, KEGG and Reactome databases (as described, [90, 91]).

## Data Availability

The MS proteomics raw data and MaxQuant search parameters have been deposited to the ProteomeXchange Consortium (http://www.proteomexchange.org/) via the PRIDE partner repository [92] with the data set identifier PXD027812.
